# Prediction of prognosis using artificial intelligence‐based histopathological image analysis in patients with soft tissue sarcomas

**DOI:** 10.1002/cam4.7252

**Published:** 2024-05-27

**Authors:** Tomohito Hagi, Tomoki Nakamura, Hiroto Yuasa, Katsunori Uchida, Kunihiro Asanuma, Akihiro Sudo, Tetsushi Wakabayahsi, Kento Morita

**Affiliations:** ^1^ Department of Orthopedic Surgery Mie University Graduate School of Medicine Tsu Japan; ^2^ Department of Oncologic Pathology Mie University Graduate School of Medicine Tsu Japan; ^3^ Department of Information Engineering Mie University Graduate School of Engineering Tsu Japan

**Keywords:** artificial intelligence, deep learning, prognosis, soft tissue sarcoma

## Abstract

**Background:**

Prompt histopathological diagnosis with accuracy is required for soft tissue sarcomas (STSs) which are still challenging. In addition, the advances in artificial intelligence (AI) along with the development of pathology slides digitization may empower the demand for the prediction of behavior of STSs. In this article, we explored the application of deep learning for prediction of prognosis from histopathological images in patients with STS.

**Methods:**

Our retrospective study included a total of 35 histopathological slides from patients with STS. We trained Inception v3 which is proposed method of convolutional neural network based survivability estimation. F1 score which identify the accuracy and area under the receiver operating characteristic curve (AUC) served as main outcome measures from a 4‐fold validation.

**Results:**

The cohort included 35 patients with a mean age of 64 years, and the mean follow‐up period was 34 months (2–66 months). Our deep learning method achieved AUC of 0.974 and an accuracy of 91.9% in predicting overall survival. Concerning with the prediction of metastasis‐free survival, the accuracy was 84.2% with the AUC of 0.852.

**Conclusion:**

AI might be used to help pathologists with accurate prognosis prediction. This study could substantially improve the clinical management of patients with STS.

## 1 INTRODUCTION

Soft tissue sarcomas (STSs) are rare malignant tumors of mesenchymal derivation that represent a heterogeneous group with many subtypes.^1^, ^2^ STSs are associated with significant morbidity and mortality and account for less than 1% of all malignancies; the incidence rate gradually increased over the last decade.^3^, ^4^, ^5^ Microscopic examination of tissue slides is an essential step in STS diagnosis.^6^ Pathologists evaluate STS from its morphological characteristics, architectural features for the diagnosis and the grading is done according to FNCLCC criteria using a microscope.^7^ It requires the pathologist to identify subtle histopathological findings in the highly complex tissue images with rapidity.^8^, ^9^

The developments in morphological examination methods have accumulated knowledge regarding the contrast between the morphological classification of tissues or cells and pathological conditions. This morphological classification was difficult to use in information processing technology because it was based on the accumulated knowledge of pathology over many years.^10^, ^11^ Since 2005, whole slide imaging (WSI) scanners have enabled the conversion of pathological specimens into digital images that can be imported into computers.^11^, ^12^

Histological grade, age, tumor size, and depth are identified as prognostic factors in patient with STS.^13^ Recently, advanced artificial intelligence (AI) techniques have demonstrated novel success in medical image analysis due to rapid progress in deep learning algorithms.^14^, ^15^, ^16^ The development of novel deep learning methods empower pathological image analysis and assist pathologists.

In the present study, we explored the application of AI in the analysis of STS pathology. We started with an overview of digital pathology, a prerequisite for the application of AI techniques. We then deeply assessed prognostic applications of machine learning in digital pathology techniques for STS. Finally, we addressed the outstanding challenges in the field and promising future directions.

## 2 PATIENTS AND METHODS

### 2.1 Patients

The medical records and histopathological slides of 35 patients who underwent surgical resection for STS were retrospectively studied. All of the surgical pathology reports and tumor paraffin blocks were collected. Samples from low grade sarcomas were excluded from this study. Clinicopathological and survival data of the patients, including age at first presentation, sex, tumor size, tumor depth, and tumor sites, were collected. The study was approved by the Institutional Review Board of Mie University Hospital. The procedures of the study were conducted in accordance with the principles of the Declaration of Helsinki. We obtained sufficient informed consent for the use of electronic patient medical records through an opt‐out strategy owing to the retrospective nature of the study.

### 2.2 Material preprocessing

**FIGURE 1:**
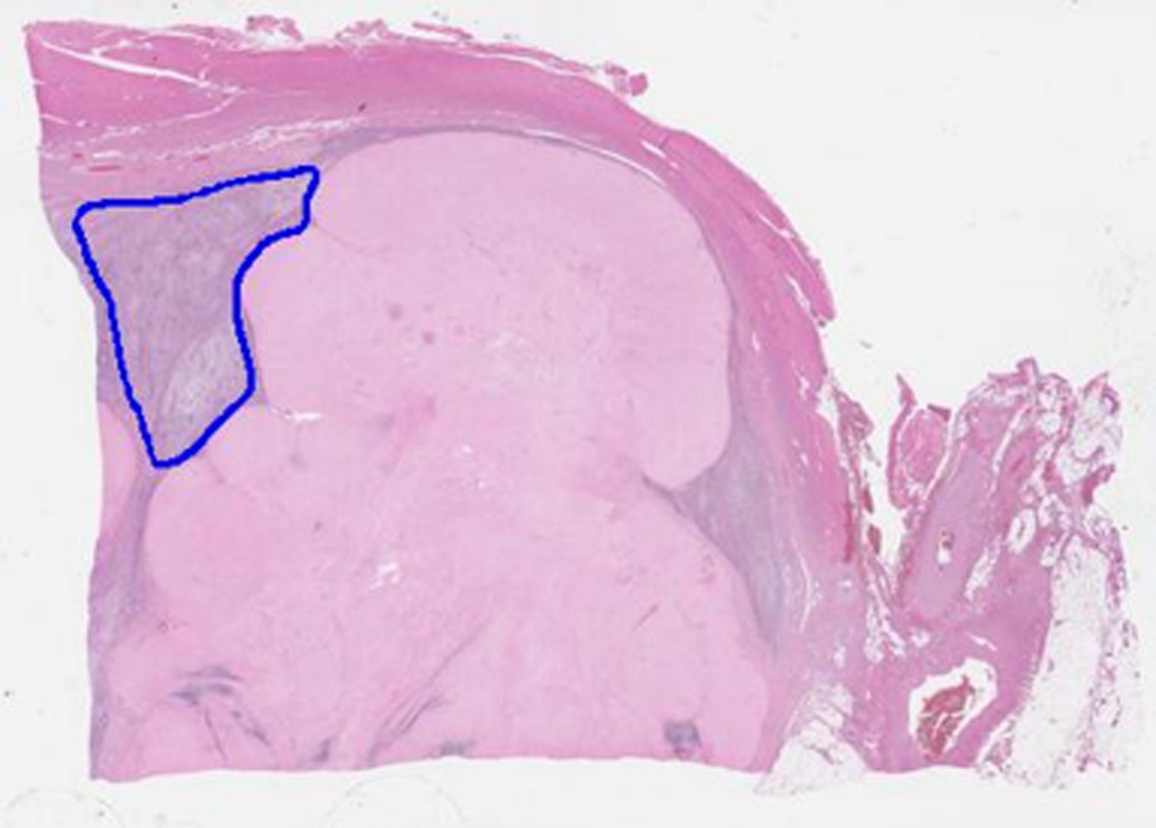
Pathological image showing hematoxylin and eosin‐stained tumor area outlined using a blue dotted line.

Hematoxylin and eosin (H & E)‐stained slides were created from paraffin blocks and then digitalized for WSI using Aperio CS2 (Leica, Tokyo, Japan) with the resolution of 20x objective. The slides were thoroughly selected and annotated by K.U. who is professional pathologist of STS and H.Y. who is also familiar to the pathology of STS, which both belong to the Department of Oncologic Pathology, Mie University Graduate School of Medicine. A total of 104,229 patches were generated from the annotations of 35 slides from 35 patients. The tumor area in the digital pathological image was outlined with a blue dotted line by pathologists which contained the highest grade of the tumor (Figure 1). There is no exclusion criteria regardless of the amount of viable tumor. The input size of WSI is relatively large compared with that of well‐known convolutional neural network models; therefore, the proposed method extracts small patches from the original image. In the patch extraction process, we use OpenSlide, a python package to handle WSI, to get pixel array and tumor area annotation by pathologist.^17^ In WSI, the sliding window strategy with a 1024 × 1024‐pixel window and 512 × 512‐pixel stride extracts approximately 10,000 patches, and the extracted patch is resized to 229 × 229 pixels to fit the input shape of Inception‐v3.^18^

### 2.3 Deep learning algorithm

For the estimation of overall and metastatic survival rates, we replaced the 1000‐unit standard Inception‐v3 output layer with a 2‐unit layer for two classes (survival or non‐survival at 3 years post resection and metastases or no metastases at 3 years post resection). The model was trained with the following parameters: The number of epochs were 300, the batch size of 16, and the optimizer was Adam with an initial learning rate of 0.0001. We trained the model entirely because most of the pre‐trained models were trained using a natural image dataset whose image characteristics were different from the original pathological images, and thus, we could collect a sufficient number of image patches owing to the size of the pathological images.^19^

The number of patients was small for numerical evaluation; therefore, a 4‐fold cross‐validation was performed. The dataset was divided into four subsets, as listed in Table 1. The results were confirmed using performance metrics, including recall (sensitivity), precision, F1 score, and area under the receiver operating characteristic curve (AUC). The F1score can be defined as a harmonic mean of the precision and recall, when the F1 score reaches its best value at 1 and worst score at 0.^20^ In each analysis, the distribution and variance of values were tested. All analyses were performed with scikit‐learn 1.2.2 and PyTorch 1.7.0 on Python 3.8.10 (Python Software Foundation, Delaware, USA).

**TABLE 1:** Datasets randomly divided into four subsets for 4‐fold cross validation.

Case number	Outcome	Estimation	Ratio
Group A			
19	1	1	0.399
8	1	1	0.001
34	0	0	0.998
14	1	1	0.323
13	0	0	0.837
4	0	0	0.995
23	1	0	0.918
25	1	1	0.006
Group B			
24	0	0	1.000
6	1	1	0.001
10	1	1	0.002
2	0	0	0.997
17	1	0	0.788
32	0	0	0.999
11	0	0	0.986
18	1	1	0.001
Group C			
12	1	1	0.000
16	0	0	0.980
1	1	1	0.005
29	1	1	0.000
22	1	1	0.000
35	0	0	0.866
3	1	1	0.416
28	0	0	0.976
30	0	0	0.722
Group D			
7	1	1	0.260
15	1	1	0.000
21	0	0	0.691
27	0	0	0.994
20	1	1	0.001
31	1	1	0.010
5	0	0	0.710
33	0	0	0.941
26	1	1	0.219
9	0	0	0.993

## 3 RESULTS

The cohort included 20 men and 15 women with a mean age of 64 years (range, 2–88 years) at the first presentation. The mean follow‐up period was 34 months (median, 36 months; range, 2–66 months). The STSs of these 35 patients were histologically classified as undifferentiated pleomorphic sarcomas (*n* = 8), dedifferentiated liposarcomas (*n* = 7), leiomyosarcomas (*n* = 6), myxofibrosarcomas (*n* = 3), synovial sarcomas (*n* = 3), myxoid liposarcomas (*n* = 2), fibrosarcoma (*n* = 2), pleomorphic liposarcoma (*n* = 1), malignant peripheral nerve sheath tumor (*n* = 1), clear cell sarcoma (*n* = 1), and rhabdomyosarcoma (*n* = 1). According to the FNCLCC histological grading system, nine sarcomas were grade 2 sarcomas, while 29 were grade 3 sarcomas (Table 2).^21^ Adjuvant radiotherapy and chemotherapy were administered to 13 and 12 patients, respectively. Adjuvant radiotherapy of irradiation dose of 50–60 Gy was considered for tumors with inadequate surgical margins. Two or three courses of doxorubicin and ifosfamide based chemotherapy were considered for tumors close to vessels, nerves, or bones. The mean tumor size was 10.7 cm (range, 4–30). Sixteen patients had tumors with a diameter >10 cm. Thirty‐three patients had tumors deep in the fascia of the underlying muscle, whereas two had superficial tumors. Thirteen patients had metastatic disease at first presentation. At the final follow‐up, eight patients were continuously disease‐free, two had no evidence of disease, two were alive with the disease, and 23 had died of the disease.

**TABLE 2:** Clinical characteristics of STS patients.

Variables	*n* = 35
Age	
Mean (years)	64
Range (years)	2–88
Sex	
Male	20 (57.1%)
Female	15 (42.9%)
Tumor size	
>10 cm	16 (45.7%)
<10 cm	19 (54.3%)
Mean (cm)	10.7
Range (cm)	4–30
Tumor site	
Extremity	20 (57.1%)
Trunk	15 (42.9%)
Tumor depth	
Superficial	2 (5.7%)
Deep	33 (94.3%)
Tumor grade	
Grade II	9 (25.7%)
Grade III	26 (74.3%)
Histological diagnosis	
Undifferentiated pleomorphic sarcoma	8 (22.9%)
Dedifferentiated liposarcoma	7 (20.0%)
Leiomyosarcoma	6 (17.1%)
Myxofibrosarcoma	3 (8.6%)
Synovial sarcoma	3 (8.6%)
Myxoid liposarcoma	2 (5.7%)
Others	6 (17.1%)
Survival Status at 3 years	
Dead	18 (51.4%)
Alive	17 (48.6%)
Metastatic survival status at 3 years	
Yes	19 (54.3%)
No	16 (45.7%)

**FIGURE 2:**
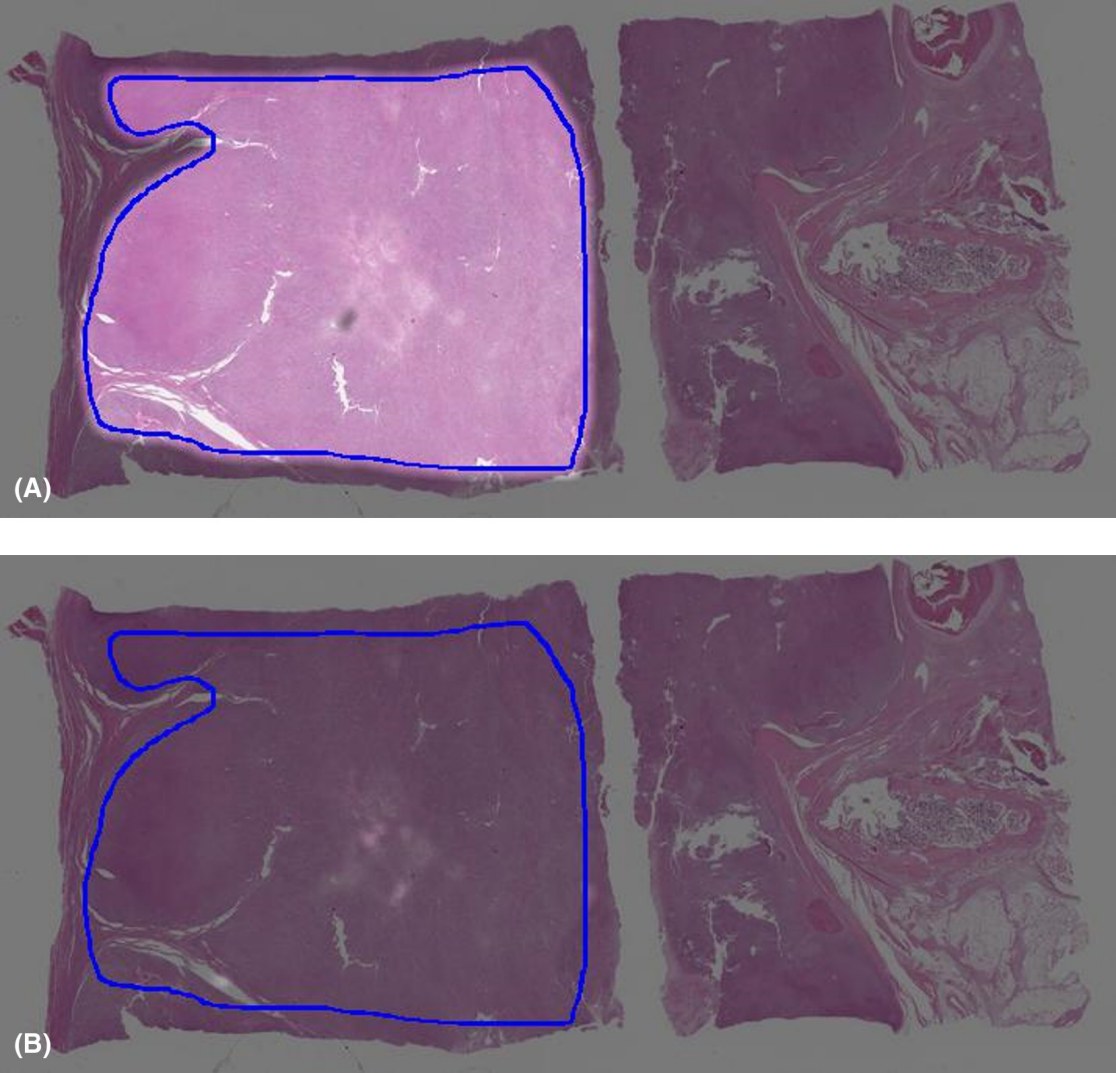
Representative heatmap for the estimation of survival obtained using whole slide imaging in a patient with dedifferentiated liposarcoma. (A) Estimation of 3‐year metastasis‐free survival, which failed. (B) Estimation of 3‐year overall survival, which failed.

**FIGURE 3:**
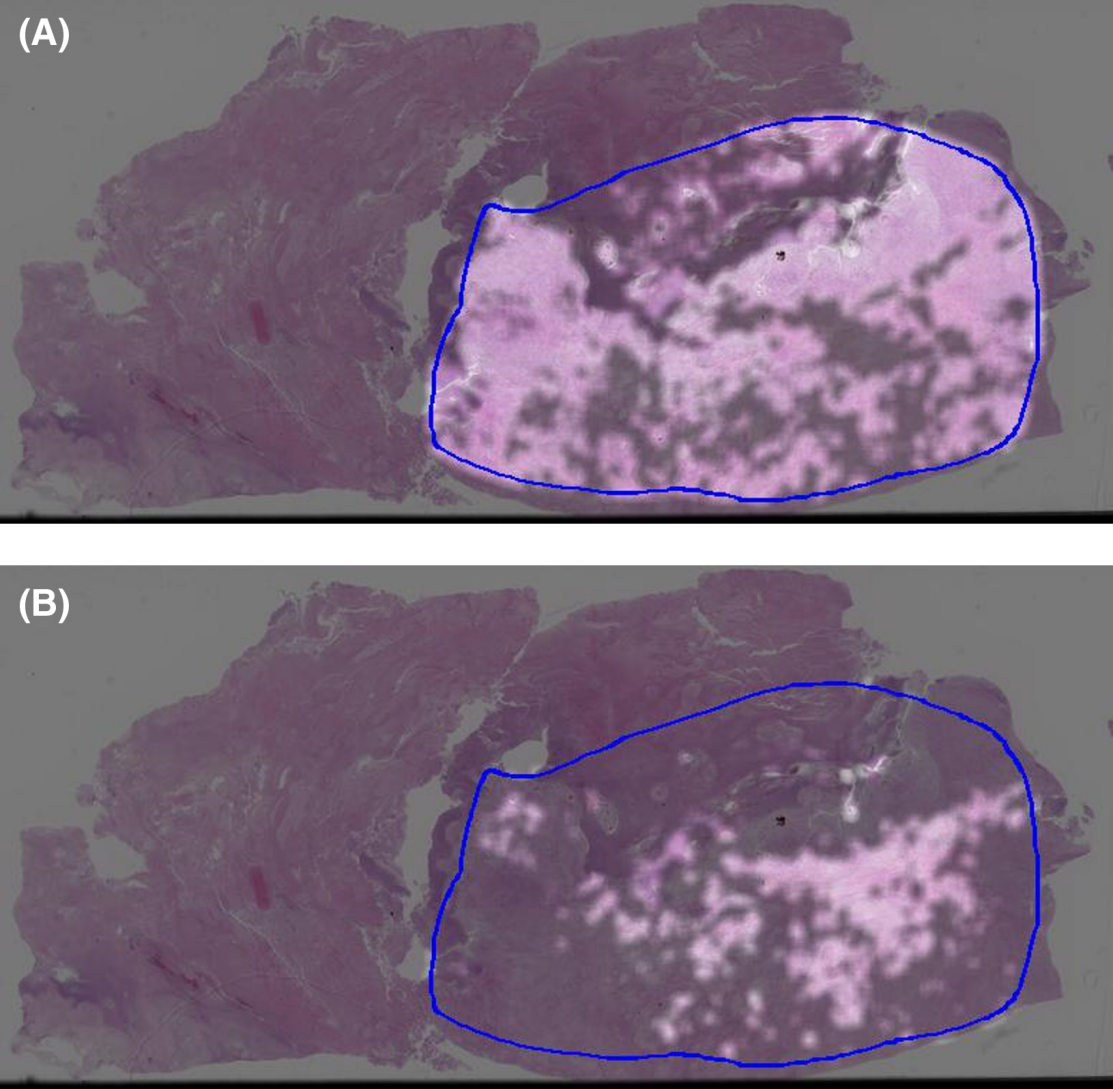
Representative heatmap for the estimation of survival obtained using whole slide imaging in a patient with myxofibrosarcoma. (A) Estimation of 3‐year metastasis‐free survival, which failed. (B) Estimation of 3‐year overall survival, which succeeded.

**FIGURE 4:**
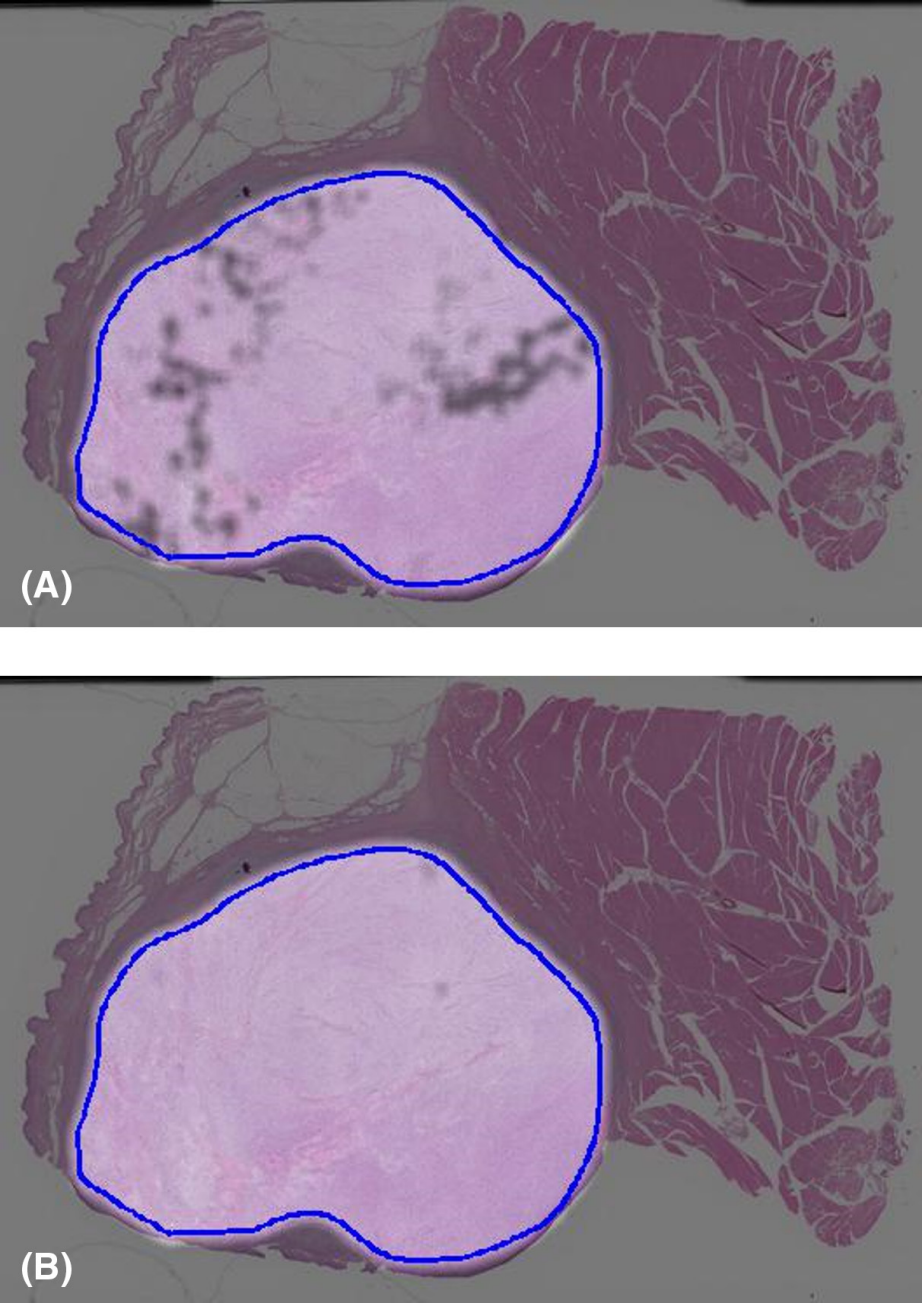
Representative heatmap for the estimation of survival obtained using whole slide imaging in a patient with undifferentiated pleomorphic sarcoma. (A) Estimation of 3‐year metastasis‐free survival, which succeeded. (B) Estimation of 3‐year overall survival, which succeeded.

A total of 104,229 patches were generated from the annotations of 35 slides from 35 patients, with a mean patch count of 2977 (±2045) per patient. The trained deep learning model estimates survival at 3 years or metastasis at 3 years in numerical values ranging from 0 to 1, which can be considered the probability of event occurrence. Figure 2 shows the event occurrence probability, where deep learning estimation was performed in the tumor area outlined using a blue line; the brighter the heatmap color, the lower the event occurrence probability. Figure 2 shows a heatmap of the whole slide image obtained from a 70‐year‐old man with dedifferentiated liposarcoma that metastasized to the lung 7 months after the initial operation and who died of the disease after 9 months after the initial surgery. Figure 2A reveals the failure of the estimation of 3‐year metastasis‐free survival, whereas Figure 2B shows that the estimation of 3‐year overall survival is successful. This patient similarly, Figure 3, shows the whole slide image obtained from a 70‐year‐old man with myxofibrosarcoma that metastasized to the lungs 4 months after the initial operation and who died of the disease after 5 months after initial surgery. Figure 3A reveals the failure of the estimation of 3‐year metastasis‐free survival, while Figure 3B shows that the estimation of 3‐year overall survival is successful; however, in both analyses, the results were borderline. On the other hand, Figure 4 shows the whole slide image obtained from a 70‐year‐old woman with undifferentiated pleomorphic sarcoma who was found to be continuously disease‐free until 5 years after initial surgery. Figure 4A,B shows that the estimations of 3‐year metastasis‐free survival and 3‐year overall survival, respectively, were successful, as represented by uniform brightness on the heatmap.

**FIGURE 5:**
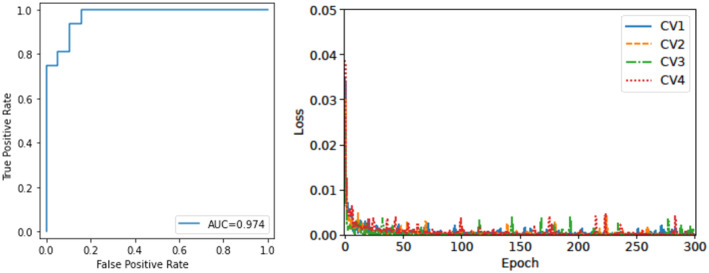
Accuracy and area under the receiver operating characteristic curve for survival prediction (left). Validation loss versus the number of epochs related to the four cross‐validation sets for survival prediction (right). AUC, area under the receiver operating characteristic curve.

**FIGURE 6:**
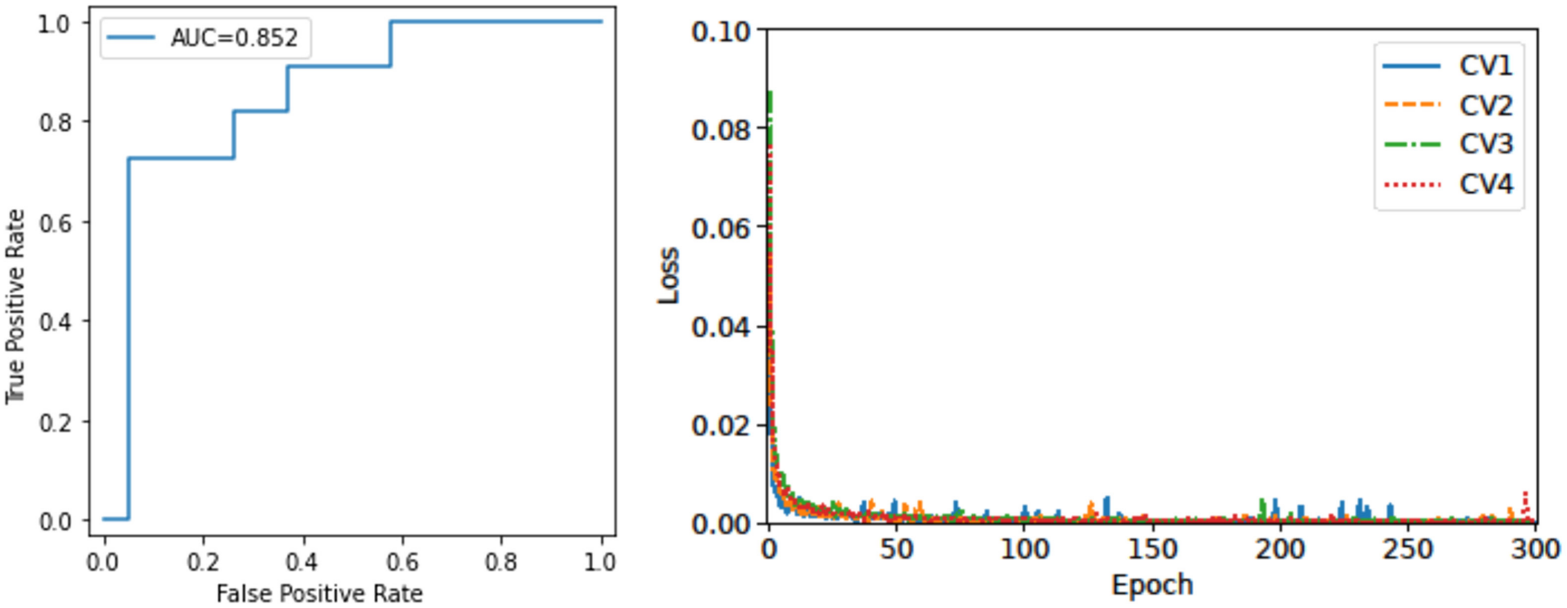
Accuracy and area under the receiver operating characteristic curve for metastasis‐free survival prediction (left). Validation loss versus the number of epochs related to the four cross‐validation sets for metastasis prediction (right). AUC, area under the receiver operating characteristic curve.

For survival or non‐survival prediction, the F1‐score was 0.919, and the AUC was 0.974 (left side of Figure 5). The recall and precision of this calculation were 0.895 and 0.944, respectively. On the right side of Figure 5 shows the validation loss versus the number of epochs for the four cross‐validation datasets. Concerning the prediction of metastasis‐free survival, the F1‐score was 0.842, and the AUC was 0.852 (left side of Figure 6). The recall and precision of this calculation were 0.842 and 0.842, respectively. The right side of Figure 6 shows the validation loss versus the number of epochs for the four cross‐validation datasets.

## 4 DISCUSSION

The advent of whole slide digital scanning and the concomitant rise in deep‐learning‐based neural networks for assessing digital images of slides have resulted in an explosion of interest in AI‐based digital pathology technologies.^15^ Despite advances in basic and translational research, STS remains a significant clinical challenge. Delay in diagnosis for patients with STS is the most common reason for complaints and litigation. The average diagnostic interval for STS is reported to be 41.2 (range, 4.3–94.6) weeks.^22^ Early detection of tumor allows more effective treatment when the tumor is at an earlier, however, this reported average diagnostic interval is quite long. Owing to the high heterogeneity and complexity of STS pathology, pathologists usually cannot diagnose STS using H & E staining alone. We believe that predicting patient outcomes only using WSI is innovative. In this current study, we presented a newly advanced approach to address this serious challenge using AI for histopathological image survival prediction in patients with STS. Furthermore, our approach can be used in small medical institutions without expert histologists.

Several reports on prognostic prediction using AI in patients with cancer exist. Kather et al. used a deep neural network to build a predictive score from histopathological images of colorectal cancer.^23^ Jiang et al. trained deep learning to combine the clinicopathological factors and computed tomography images to predict the survival of patients with stomach cancer.^24^ These reports describe multimodal fusion models that integrate histopathological images and other omics data to improve prognostic accuracy. In contrast, in the present study, we developed a novel deep‐learning approach using only histopathological data, with a high accuracy rate of 91.9% and an AUC of 0.974 for overall survival prognosis and 84.2% and an AUC of 0.852 for metastasis‐free survival prognosis. We used 4‐fold cross‐validation, a common method for effectively improving model robustness to assess model performance.^25^

Although we developed an automated assistance method to predict patient survival, one major limitation of this study was using a single validation dataset from our institution. Future studies should investigate other institutional datasets for validation purposes. Because the H & E staining method differs subtly among institutions, evaluating several datasets could increase our model's credibility.

## 5 CONCLUSION

We conclude that our deep learning method might be used to help pathologists with accurate prognosis prediction. The novel method accurately predicted both overall and metastasis‐free survival. This could substantially improve the clinical management of patients with STS. However, this is a pilot study with small sample of size and so requires further investigation with different validation datasets and also each individual STS.

## AUTHOR CONTRIBUTIONS

**Tomohito Hagi:** Conceptualization (equal); data curation (lead); formal analysis (lead); funding acquisition (lead); investigation (lead); methodology (equal); project administration (lead); software (equal); supervision (equal); validation (equal); visualization (equal); writing – original draft (lead). **Tomoki Nakamura:** Conceptualization (lead); data curation (equal); formal analysis (equal); methodology (equal); project administration (equal); resources (equal); supervision (lead); writing – review and editing (lead). **Hiroto Yuasa:** Investigation (equal); methodology (equal); resources (equal); validation (equal); visualization (equal). **Katsunori Uchida:** Investigation (equal); methodology (equal); supervision (equal); validation (equal). **Kunihiro Asanuma:** Conceptualization (equal); data curation (equal); investigation (equal); supervision (equal). **Akihiro Sudo:** Supervision (equal); writing – review and editing (equal). **Tetsushi Wakabayahsi:** Investigation (equal); project administration (equal); supervision (equal). **Kento Morita:** Conceptualization (equal); investigation (equal); methodology (equal); software (equal); validation (equal); writing – original draft (supporting).

## FUNDING INFORMATION

This study was supported by a Grant of the Japan Orthopaedics and Traumatology Foundation (No. 462).

## CONFLICT OF INTEREST STATEMENT

The authors declare that they have no known competing financial interests or personal relationships that could have appeared to influence the work reported in this paper.

## Data Availability

The datasets generated and analyzed during the current study are available from the corresponding author on reasonable request.
